# Demographic and Clinical Characteristics Associated with Engagement in Behavioral Health Treatment Among Children with Autism Spectrum Disorders

**DOI:** 10.1007/s10803-017-3247-5

**Published:** 2017-07-26

**Authors:** Lisa A. Croen, Naomi Shankute, Meghan Davignon, Maria L. Massolo, Cathleen Yoshida

**Affiliations:** 1 Division of Research, Kaiser Permanente Northern California, 2000 Broadway, Oakland, CA 94612 USA; 2 Kaiser Roseville Medical Center, Pediatric Specialties, Roseville, CA USA; 3 Present Address: Quality and Operations Support, Kaiser Permanente California, Oakland, CA 94612 USA

**Keywords:** Applied behavior analysis (ABA), intervention, Treatment adherence, Demographic factors

## Abstract

This study investigates demographic and clinical factors associated with initiation, continuation, and adherence to behavioral health treatment (BHT) among children with autism spectrum disorder. Among 293 insured children referred for applied behavior analysis (ABA) based BHT, 23% never initiated treatment. Among those initiating treatment, 31% discontinued treatment within 1 year of treatment initiation, and only 15% received 80% or more of recommended treatment hours. Younger age at referral to treatment, private health insurance, and receiving more than 10 h/week of BHT were associated with treatment engagement. Co-occurring psychiatric and medical conditions were related to treatment discontinuation among children 5 years or older. These findings suggest specific subgroups that may benefit from additional support with engaging in recommended behavioral health treatment.

## Introduction

Autism spectrum disorders (ASD) are complex neurodevelopmental disorders marked by restricted communication, difficulties with social interactions and repetitive behaviors (American Psychiatric Association 2013). The prevalence of ASD is high, with 1 in 68 children aged 8 identified with ASD in the United States (Christensen et al. 2016). If left untreated the core features associated with ASD may negatively impact the individual, including less likelihood of mainstreaming in school, increased likelihood of out of home placement, and behavioral, feeding, and sleeping problems, which in turn can negatively impact the family unit (Fenske et al. 1985; Kodak and Piazza 2008).

BHT—intervention using evidence based principles such as those used in ABA—has been shown to ameliorate some of the symptoms associated with ASD (Jensen and Sinclair 2002; Granpeesheh et al. 2009; Reichow et al. 2012). BHT rooted in ABA has a large evidence base and is effective in improving a wide range of outcomes including full-scale IQ (intelligence quotient), receptive and expressive language, and social, daily living, adaptive, and communication skills (Howard et al. 2005; Eldevik et al. 2009; Virues-Ortega 2010; Reichow et al. 2012; MacDonald et al. 2014). Additionally, studies have shown maintenance of outcomes well beyond the termination of interventions (Rogers 1996; Virues-Ortega 2010). Behavioral intervention can also ameliorate some common co-occurring conditions in children with ASD, including sleep and feeding disorders as well as pica (a disorder marked by ingesting inedible substances) (Kodak and Piazza 2008).

As a consequence of autism insurance legislation enacted in 2012 in California (SB946), BHT is now a covered treatment for most insured patients with ASD. In order for individuals to benefit, treatment programs must be initiated and followed. If initiation, continuation, and adherence to behavioral treatment services do not occur, patients may miss out on potential social, communication, and behavior gains. Structured approaches to developing ABA treatment plans informed by patient and family characteristics could improve treatment initiation, continuation, and adherence among patients with ASD (Kasari 2002; Green et al. 2006; Stahmer et al. 2011; Fava and Strauss 2014; Vivanti et al. 2014). However, there is limited information about the specific factors that facilitate or inhibit the initiation and continuation of ABA behavioral therapy among children with ASD.

There have been studies identifying factors affecting treatment initiation for other pediatric chronic conditions, such as mental health conditions. Some characteristics identified as barriers to treatment attendance and participation in children’s psychosocial therapy include lower socioeconomic status and income level, ethnic minority status, single-parent household, and older child’s age (Nock and Kazdin 2001). Factors such as occupation, education, income, and family composition have been associated with premature termination of child mental health services among Black families when compared to White families (Kazdin et al. 1995).

The objective of this study was to identify factors that are associated with initiation, continuation, and adherence to BHT in a population of insured children with ASD who were referred for BHT treatment. Results from this investigation will inform providers of facilitators and barriers to BHT and aid in the development of strategies to identify individuals who may benefit from additional resources or support.

## Methods

We conducted a cross-sectional study comprised of children aged 2–18 years old with ASD who were referred for BHT between February and May 2014. All children were insured by Kaiser Permanente in Northern California (KPNC), the largest integrated health care delivery system in the United States. KPNC insures over 3.9 million children and adults residing in the San Francisco and Sacramento metropolitan areas and surrounding counties. KPNC members are largely representative of the population residing in the counties served by the health plan with respect to sociodemographic and clinical characteristics (Krieger 1992).

KPNC contracts BHT services with vendors in the community. Once a child is referred for BHT, the first step is for the child to undergo an assessment by the BHT vendor in order to establish a treatment plan, including the number of recommended hours of treatment. Since our focus was on factors related to initiating ABA treatment, only patients with no previous assessment for, or receipt of BHT prior to their referral date were eligible for inclusion (N = 308). We excluded patients with referrals canceled by KPNC (N = 15) due to ineligibility (e.g., no longer insured), resulting in a final analysis cohort of 293 patients.

### Outcome Definitions

Patients were assigned to 1 of 4 outcome categories: Group 1, “never assessed and never started treatment,” comprised of patients who had no billing for an assessment visit and no claims for behavioral treatment; Group 2, “assessed but never started treatment,” consisted of patients who had been billed for an assessment but had no claims for behavioral treatment; Groups 3 and 4 consisted of patients who were both assessed for treatment and initiated treatment. Group 3, “initiated but discontinued treatment,” consisted of patients who received at least two consecutive weeks of BHT treatment delivered by a behavioral interventionist, program supervisor, or case manager (all members of the treatment delivery team), but had more than three consecutive months of no treatment within the 12-month period following the start of treatment. Group 4, “initiated and continued treatment,” consisted of patients who stayed in treatment for 12 months with no gaps in treatment of 3 months or more. Among patients who initiated treatment, treatment adherence was defined as the delivery of 80% or more of the authorized hours.

### Predictors

We obtained data on several patient socio-demographic factors and clinical characteristics from KPNC electronic medical records. Sociodemographic factors included child sex, race/ethnicity (White, Hispanic, Asian, and Black/Other), age at referral to BHT (<5, ≥5), born at KP hospital (Yes/No), type of health insurance (employer/self-funded, state subsidized), simplex/multiplex status (multiplex defined as having a sibling with ASD), copay amount (>$20, ≤$20), maternal age at referral to BHT, and maternal and paternal education at referral to BHT (≤high school, college/post-graduate). Child clinical characteristics included medical complexity [number of co-occurring medical and/or mental health conditions: (0–1, ≥2)], psychiatric medication use prior to referral to BHT (Yes/No); and receipt of speech/language therapy, occupational therapy, or physical therapy at time of referral to BHT (Yes/No).

### Statistical Analytic Approach

We conducted bivariate and multivariate analyses to explore the association between each socio-demographic and clinical factor and the binary outcomes of interest: initiating treatment (Groups 1 + 2 vs. Groups 3 + 4), continuing treatment (Group 3 vs. Group 4), and treatment adherence (Group 3 vs. Group 4). We used Pearson’s chi-squared test for categorical variables and Wilcoxon rank sum tests for non-normal continuous variables. We considered results statistically significant if they had p values < 0.05. We fit separate multivariate logistic regression models for each outcome of interest to estimate the independent effects of each sociodemographic and clinical factor after controlling for all other variables in the model. Multivariate analyses were restricted to records with non-missing values for each covariate included in the model.

This study was approved by the Kaiser Permanente Institutional Review Board.

## Results

Characteristics of the study population are shown in Table 1. The median age at referral to BHT was 6.5 years. The majority of patients referred for treatment were male, born in KPNC, had employer or self-funded insurance, had copay amounts of 20 dollars or less, were the only child in the household with an ASD diagnosis, and had parents older than 30 years of age with more than a high school education. Additionally, nearly half had two or more medical comorbidities, the most common being gastrointestinal (GI) disorders and psychiatric disorders, and approximately 15–25% had a history of psychiatric medications, received speech therapy, physical therapy and/or occupational therapy at the time of referral for ABA (Table 1).

**Table 1:** Characteristics of the study population, kaiser permanente members referred for behavioral health therapy (BHT), 2014

Characteristic	Total (N = 293)	Group 1 Not assessed (N = 49)	Group 2 Assessed but did not initiate treatment (N = 18)	Group 3 Initiated but discontinued treatment (N = 70)	Group 4 Initiated and continued treatment (N = 156)
	n (%)	n (%)	n (%)	n (%)	n (%)
Child median age at referral for treatment, years (SD)	6.5 (4.1)	8.2 (4.4)	8.3 (4.4)	7.3 (4.1)	6.0 (3.8)
Child age at referral for treatment, years					
<5	105 (36)	13 (27)	5 (28)	23 (33)	64 (41)
5–<12	139 (47)	25 (51)	10 (56)	32 (46)	72 (46)
≥12	49 (17)	11 (22)	3 (17)	15 (21)	20 (13)
Child’s race/ethnicity					
White	108 (37)	20 (41)	5 (28)	29 (41)	54 (35)
Hispanic	65 (22)	9 (18)	6 (33)	18 (26)	32 (21)
Asian	78 (27)	11 (22)	4 (22)	16 (23)	47 (30)
Black/other	42 (14)	9 (18)	3 (17)	7 (10)	23 (15)
Sex					
Male	244 (83)	41 (84)	15 (83)	61 (87)	127 (81)
Female	49 (17)	8 (16)	3 (17)	9 (13)	29 (19)
Born in Kaiser					
Yes	173 (59)	28 (57)	7 (39)	47 (67)	91 (58)
No	120 (41)	21 (43)	11 (61)	23 (33)	65 (42)
Parent insurance coverage type^a^					
Employer/self-funded	272 (93)	43 (90)	14 (78)	64 (91)	151 (97)
State subsidized	20 (7)	5 (10)	4 (22)	6 (9)	5 (3)
Child’s copay for BHT^a^					
≤$20	218 (77)	32 (73)	12 (80)	57 (84)	117 (75)
>$20	64 (23)	12 (27)	3 (20)	11 (16)	38 (25)
Multiple ASD in family^a^					
Yes	34 (12)	7 (15)	0 (0)	11 (16)	16 (10)
No	257 (88)	40 (85)	18 (100)	59 (84)	140 (90)
Mother median age at child’s referral for treatment, years (SD)	38.2 (7.2)	38.1 (7.0)	36.8 (10.2)	37.6 (7.4)	38.6 (6.8)
Mother age at child’s referral for treatment, years^a^					
<30	26 (10)	2 (5)	5 (31)	9 (14)	10 (7)
30–39	130 (48)	21 (51)	4 (25)	29 (44)	76 (51)
≥40	116 (43)	18 (44)	7 (44)	28 (42)	63 (42)
Mother education^a^					
≤ High school graduate	47 (26)	8 (24)	6 (67)	11 (22)	22 (24)
Undergraduate/Postgraduate	137 (74)	25 (76)	3 (33)	39 (78)	70 (76)
Father median age at child’s referral for treatment, years (SD)^a^	41.3 (7.9)	41.2 (7.5)	39.3 (8.6)	42.0 (7.8)	–
Father age at child’s referral for treatment ^a^					
<30	14 (6)	3 (8)	1 (8)	6 (12)	4 (3)
30–39	87 (37)	12 (32)	5 (42)	16 (31)	54 (41)
≥40	134 (57)	23 (61)	6 (50)	30 (58)	75 (56)
Father education^a^					
≤ High school graduate	53 (29)	11 (33)	4 (50)	20 (42)	18 (20)
Undergraduate/Postgraduate	127 (71)	22 (67)	4 (50)	28 (58)	73 (80)
Psychiatric comorbidities					
Yes	81 (28)	19 (39)	4 (22)	29 (41)	29 (19)
No	212 (72)	30 (61)	14 (78)	41 (59)	127 (81)
Intellectual disability					
Yes	29 (10)	2 (4)	4 (22)	3 (4)	20 (13)
No	264 (90)	47 (96)	14 (78)	67 (96)	136 (87)
Sleep problems					
Yes	14 (5)	3 (6)	1 (6)	6 (9)	4 (3)
No	279 (95)	46 (94)	17 (94)	64 (91)	152 (97)
Seizure disorder/seizure					
Yes	12 (4)	0 (0)	1 (6)	2 (3)	9 (6)
No	281 (96)	49 (100)	17 (94)	68 (97)	147 (94)
GI disorder					
Yes	135 (46)	21 (43)	9 (50)	39 (56)	66 (42)
No	158 (54)	28 (57)	9 (50)	31 (44)	90 (58)
Genetic syndrome					
Yes	21 (7)	2 (4)	2 (11)	11 (16)	6 (4)
No	272 (93)	47 (6)	16 (89)	59 (84)	150 (96)
Medical complexity					
ASD with 0–1 co-occurring conditions	159 (54)	27 (55)	9 (50)	34 (49)	89 (57)
ASD with ≥2 co-occurring conditions	134 (46)	22 (45)	9 (50)	36 (51)	67 (43)
Psychiatric medications					
Stimulants/ADHD Meds					
Yes	43 (15)	10 (20)	3 (17)	13 (19)	17 (11)
No	250 (85)	39 (80)	15 (83)	57 (81)	139 (89)
Antipsychotic 2nd generation					
Yes	22 (8)	5 (10)	1 (6)	4 (6)	12 (8)
No	271 (92)	44 (90)	17 (94)	66 (94)	144 (92)
Antidepressants					
Yes	24 (8)	5 (10)	1 (6)	10 (14)	8 (5)
No	269 (92)	44 (90)	17 (94)	60 (86)	148 (95)
Anticonvulsant					
Yes	8 (3)	0 (0)	1 (6)	3 (4)	4 (3)
No	285 (97)	49 (100)	17 (94)	67 (96)	152 (97)
Antianxiety					
Yes	15 (5)	2 (4)	1 (6)	2 (3)	10 (6)
No	278 (95)	47 (96)	17 (94)	68 (97)	146 (94)
Benzodiazepine					
Yes	20 (7)	3 (6)	1 (6)	7 (10)	9 (6)
No	273 (93)	46 (94)	17 (94)	63 (90)	147 (94)
Any psychiatric medications					
Yes	78 (27)	17 (35)	4 (22)	22 (31)	35 (22)
No	215 (73)	32 (65)	14 (78)	48 (69)	121 (78)
KP therapies received at the time of referral for BHT					
Speech/Language therapy					
Yes	41 (14)	3 (6)	3 (17)	8 (11)	27 (17)
No	252 (86)	46 (94)	15 (83)	62 (89)	129 (83)
Occupational therapy/physical therapy					
Yes	39 (13)	6 (12)	3 (17)	8 (11)	22 (14)
No	254 (87)	43 (88)	15 (83)	62 (89)	134 (86)
Feeding therapy					
Yes	4 (1)	1 (2)	0 (0)	0 (0)	3 (2)
No	289 (99)	48 (98)	18 (100)	70 (100)	153 (98)

*SD* standard deviation, *ADHD* attention deficit hyperactivity disorder, *BHT* behavioral health treatment

^a^Numbers don’t add up to column totals due to missing data

Among the 23% of patients (N = 67) who did not initiate treatment (Groups 1 and 2), 73% (Group 1, N = 49) never completed the first step, which was an in-person assessment by the treatment provider. Among the 77% (N = 226) who initiated treatment (Groups 3 and 4), 31% (Group 3, N = 70) discontinued treatment within 12 months of treatment initiation.

### Initiation of Treatment

Patients who initiated treatment were significantly younger at referral (median 6.3 years vs. 8.2 years, p = 0.02), and less likely to have state subsidized health insurance (5 vs. 14%, p = 0.01) than patients who did not initiate treatment. Maternal education level was significantly lower among those with state subsidized health insurance than employer/self-funded insurance (≤high school: 47 vs. 23%, p = 0.04). Among children 5 years or older of age initiation of treatment was more common among those whose mothers were older than 30 years of age at referral of child (98 vs. 88%, p = 0.001). In adjusted analyses (Table 2), state subsidized insurance was the only factor that was significantly associated with not initiating BHT (adjusted odds ratio OR_a_ 0.3, 95% CI [0.1–0.9]). Children who initiated BHT were similar to children who did not initiate BHT with respect to clinical characteristics, including age at ASD diagnosis, number of co-occurring medical conditions, and receipt of other therapies including psychotropic medications, speech/language therapy, and physical/occupation therapy (Table 3).

**Table 2:** Treatment initiation by demographic characteristics

Characteristic	Initiated treatment (Groups 3 + 4) (N = 223)	Did not initiate treatment (Groups 1 + 2) (N = 58)	Crude OR	95% CI	Adjusted OR	95% CI
	n (%)	n (%)				
Child sex						
Male	186 (83)	49 (84)	1	Reference	1	Reference
Female	37 (17)	9 (16)	1.1	[0.5–2.4]	1	[0.4–2.3]
Race/ethnicity						
White	82 (37)	22 (38)	1	Reference	1	Reference
Hispanic	49 (22)	13 (22)	1	[0.5–2.2]	1.1	[0.5–2.6]
Asian	63 (28)	13 (22)	1.3	[0.6–2.8]	1.2	[0.5–2.6]
Black/other	29 (13)	10 (17)	0.8	[0.3–1.8]	0.8	[0.3–2.0]
Child age at referral median, years (SD)	6.3 (3.9)	7.7 (4.2)	0.9	[0.9–1.0]	0.9	[0.9–1.0]
Born in Kaiser						
Yes	136 (61)	33 (57)	1.2	[0.7–2.1]	1.1	[0.6–2.1]
No	87 (39)	25 (43)	1	Reference	1	Reference
Multiple ASD in family						
Yes	27 (12)	7 (12)	1	[0.4–2.4]	1	[0.4–2.5]
No	196 (88)	51 (88)	1	Reference	1	Reference
Insurance coverage type						
State subsidized	11 (5)	8 (14)	0.3	[0.1–0.8]^a^	0.3	[0.1–0.9]^a^
Employer/self-funded	212 (95)	50 (86)	1	Reference	1	Reference
Copay for BHT						
≤$20	174 (78)	44 (76)	1	Reference	1	Reference
>$20	49 (22)	14 (24)	0.9	[0.4–1.7]	0.8	[0.4–1.5]

*SD* standard deviation, *OR* odds ratio, *CI* confidence interval

Adjusted for all variables in the table

^a^Indicates that the odds ratio is statistically significant

**Table 3:** Treatment initiation by child clinical characteristics

Characteristic	Initiated treatment (Groups 3 + 4) (N = 226)	Did not initiate treatment (Groups 1 + 2) (N = 67)	Crude OR	95% CI	Adjusted OR	95% CI
	n (%)	n (%)				
Child’s age at ASD diagnosis median (SD)	5.6 (3.7)	5.9 (4.3)	0.9	[0.9–1.00]	0.9	[0.9–1.0]
Medical complexity						
ASD with 0–1 co-occurring conditions	123 (54)	36 (54)	1	Reference	1	Reference
ASD ≥ 2 co-occurring conditions	103 (46)	31 (46)	1	[0.6–1.7]	0.9	[0.5–1.7]
Any psychiatric medications						
Yes	57 (25)	21 (31)	0.7	[0.4–1.3]	0.9	[0.5–1.8]
No	169 (75)	46 (69)	1	Reference	1	Reference
Speech/language therapy						
Yes	35 (15)	6 (9)	1.9	[0.7–4.6]	1.7	[0.7–4.6]
No	191 (85)	61 (91)	1	Reference	1	Reference
Occupational therapy/physical therapy						
Yes	30 (13)	9 (13)	1	[0.4–2.2]	0.8	[0.4–2.0]
No	196 (87)	58 (87)	1	Reference	1	Reference

Adjusted for all variables in the table

*SD* standard deviation, *OR* odds ratio, *CI* confidence interval

### Continuation of Treatment

In bivariate analyses of demographic characteristics, children who initiated and continued treatment were significantly younger at referral than children who initiated but discontinued treatment (median age: 6.0 years vs. 7.3 years, p = 0.04). In addition, treatment continuation was significantly associated with father’s age older than 30 years (97 vs. 88%, p = 0.05) and fathers with education beyond high school (80 vs. 58%, p = 0.006); however data on paternal age and education was only available for 82 and 62% of the study population, respectively. Among children younger than 5 years of age, treatment continuation was significantly associated with maternal age older than 30 years (86 vs. 61%, p = 0.03). Among children 5 years or older, treatment continuation was associated with higher co-pay (>$20: 29 vs. 11%, p = 0.02). In adjusted analyses, child age at referral for BHT was the only demographic characteristic that was significantly associated with continuation of treatment in the total group of children (Table 4).

**Table 4:** Treatment continuation by demographic characteristics

Characteristic	Initiated and continued treatment (Group 4) (N = 155)	Initiated and discontinued treatment (Group 3) (N = 68)	Crude OR	95% CI	Adjusted OR	95% CI
	n (%)	n (%)				
Child sex						
Male	127 (82)	59 (87)	1	Reference	1	Reference
Female	28 (18)	9 (13)	1.4	[0.6–3.3]	1.5	[0.6–3.5]
Child race/ethnicity						
White	54 (35)	28 (41)	1	Reference	1	Reference
Hispanic	31 (20)	18 (26)	0.9	[0.4–1.9]	1	[0.4–2.1]
Asian	47 (30)	16 (24)	1.5	[0.7–3.2]	1.2	[0.6–2.6]
Black/Other	23 (15)	6 (9)	2	[0.7–5.4]	1.8	[0.6–5.2]
Child age at referral for BHT median, years (SD)	6.0 (3.8)	7.3 (4.1)	0.9	[0.9–1.0]^a^	0.9	[0.9–1.0]^a^
Born in Kaiser						
Yes	91 (59)	45 (66)	0.7	[0.4–1.3]	0.7	[0.4–1.3]
No	64 (41)	23 (34)	1	Reference	1	Reference
Multiple ASD in family						
Yes	16 (10)	11 (16)	0.6	[0.3–1.4]	0.7	[0.3–1.6]
No	139 (90)	57 (84)	1	Reference	1	Reference
Insurance coverage type						
State Subsidized	5 (3)	6 (9)	0.3	[0.1–1.2]	0.4	[0.1–1.5]
Employer/Self Funded	150 (97)	62 (91)	1	Reference	1	Reference
Copay for behavioral health treatment						
≤$20	117 (75)	57 (84)	1	Reference	1	Reference
>$20	38 (25)	11 (16)	1.7	[0.8–3.5]	1.4	[0.7–3.1]

Adjusted for all variables in the table

*SD* standard deviation, *OR* odds ratio, *CI* confidence interval

^a^Indicates that the odds ratio is statistically significant

With respect to clinical characteristics, bivariate analyses indicated that children who continued BHT had a shorter interval between treatment authorization and treatment initiation (<4 weeks: 80 vs. 69%, p = 0.05), and were less likely to have psychiatric comorbidity (19 vs. 41%, p < 0.01), or a genetic syndrome (4 vs. 16%, p < 0.01) than children who discontinued treatment. Among the subset of children referred to BHT at 5 years or older, psychiatric conditions (32 vs. 57%, p = 0.003), sleep problems (2 vs. 11%, p = 0.04), genetic syndromes (4 vs. 21%, p = 0.005), and use of antidepressant/anti-anxiety medications (9 vs. 21%, p = 0.04) were less common among those who continued treatment compared with those who discontinued treatment. In adjusted analyses, children 2–18 years of age who continued treatment were more likely to receive more than 10 h of BHT a week than patients who discontinued treatment (Table 5). There was no difference between patients who continued and discontinued BHT with respect to medical complexity, and receipt of other therapies including psychiatric medications, speech and language therapy, and occupational and physical therapy in adjusted analyses (Table 5).

**Table 5:** Treatment continuation by child clinical characteristics

Characteristic	Initiated and continued treatment (Group 4) (N = 152)	Initiated and discontinued treatment (Group 3) (N = 65)	Crude OR	95% CI	Adjusted OR	95% CI
	n (%)	n (%)				
Child’s age at ASD diagnosis median, years (SD)	5.1 (3.4)	6.6 (3.9)	0.9	[0.8–1.0]	1	[0.9–1.1]
Medical complexity						
ASD with 0–1 co-occurring conditions	88 (58%)	32 (49%)	1	Reference	1	Reference
ASD ≥ 2 co-occurring conditions	64 (42%)	33 (51%)	0.7	[0.4–1.3]	0.7	[0.4–1.3]
Any psychiatric medications						
Yes	32 (21%)	20 (31%)	0.6	[0.3–1.2]	0.9	[0.4–2.0]
No	120 (79%)	45 (69%)	1	Reference	1	Reference
Speech/language therapy						
Yes	27 (18%)	8 (12%)	1.5	[0.7–3.6]	1.8	[0.7–4.6]
No	125 (82%)	57 (88%)	1	Reference	1	Reference
Occupational therapy/physical therapy						
Yes	20 (13%)	8 (12%)	1.1	[0.4–2.6]	1	[0.4–2.7]
No	132 (87%)	57 (88%)	1	Reference	1	Reference
Total number of ABA hours per week (behavioral interventionist, program supervisor, case manager)						
<10	78 (51%)	53 (82%)	1	Reference	1	Reference
≥10	74 (49%)	12 (18%)	4.3	[2.1–8.6]^a^	3.4	[1.5–7.4]^a^
Adherent						
<0.8	124 (82%)	60 (92%)	1	Reference	1	Reference
≥0.8	28 (18%)	5 (8%)	2.7	[1.0–7.4]	1.6	[0.6–4.8]

Adjusted for all variables in the table

*SD* standard deviation, *OR* odds ratio, *CI* confidence interval

^a^Indicates that the odds ratio is statistically significant

### Treatment Adherence

Among patients who initiated treatment, only 15% (33/220) received 80% or more of the authorized hours of treatment and were considered adherent. Characteristics of patients that were associated with receiving at least 80% of authorized hours included younger median age at referral to BHT (4.5 years vs. 6.3 years, p = 0.08) and receipt of 10 or more h/week of BHT (76 vs. 38%, p < 0.01).

## Discussion

In this large population of insured children with ASD, we found that nearly 25% who were referred for BHT never initiated treatment, and among those who initiated treatment, approximately 30% discontinued treatment within 12 months. These findings are similar to previous reports looking at treatment for children for psychopathology in general, which found that 40–60% of families never attend their first session (Gopalan et al. 2010) or drop-out prior to treatment completion (Kazdin 1995; Gopalan et al. 2010). Additionally, our findings are similar to previous studies of ASD-specific behavioral interventions. One study looking at treatment attendance among low-resourced children with ASD enrolled in a randomized controlled trial targeting core deficits of ASD found that 20% dropped out of the trial before treatment started (Carr et al. 2016).

We found that only 15% of families who initiated and continued therapy for at least 12 months were adherent to treatment (≥80% of authorized treatment hours delivered). There is limited information on the engagement of patients with ASD in BHT in community-based treatment samples; rates of behavioral treatment engagement in general have not been examined systemically in the field of ASD (Carr et al. 2016). In their randomized controlled trial, Carr et al. (2016) reported that among families who did engage in treatment, 41% had poor attendance (<70% of sessions attended). While reasons for missed sessions were not specifically obtained for this treatment population, results from prior studies (Carr and Lord 2016) and interviews with a similar population within KPNC (data unpublished) indicate the reasons are manifold and include parent or child illness, conflicts with parent work schedule or other behavioral or medical appointments for the child, difficulties with transportation, and issues related to the intervention team including appointment cancellations, staff turnover, and lack of available behavioral interventionists to administer treatment.

Age at referral was the strongest predictor of BHT treatment initiation, continuation, and adherence. Younger patients were more likely to initiate and continue treatment services, consistent with previous research (Thomas et al. 2007). Initiation at a younger age may be a result of increased awareness on the part of parents of the benefits of early intervention for children with ASD and their push for earlier diagnosis and treatment. Parents of younger children may also have more motivation and time to pursue BHT because their young child may not yet be in school and thus may not be receiving any services through the educational system.

While we did not have information on household income, we used insurance type as a proxy for socioeconomic status. We found that health insurance type was associated with BHT initiation. Patients with private insurance (self-pay or employer-funded) were more likely to initiate BHT than those with public insurance. Our findings are consistent with Nguyen et al. (2016), who reported that children with public insurance received significantly less hours of individual services than those with private insurance. Our findings are also consistent with Thomas et al. (2007), who reported that families with higher income levels were more likely to use services for ASD. Irvin et al. (2012) also reported that caregivers with higher socioeconomic status were more likely to enroll their children in ABA services. In our study population, the families with private insurance may have more resources than those with state subsidized insurance. Additionally they may be better able to afford deductibles and co-pays which can be considerable for children receiving BHT services several times a week.

Our study did not find significant differences in initiation or continuation of treatment related to race/ethnicity. This finding contrasts with prior research which indicated that belonging to a racial or ethnic minority group contributed to difficulty accessing services for ASD (Thomas et al. 2007; Liptak et al. 2008), including individual-based services (Nguyen et al. 2016). The reasons for this discrepancy are not known, but may be related to differences between study populations in insurance coverage (in our study, all children had health insurance) and access to treatment services.

We found that a shorter interval between assessment for treatment and treatment initiation was associated with treatment continuation. Likewise, children with more treatment hours delivered per week were more likely to continue treatment. It is possible that families who are able to move more quickly through the process from referral to treatment initiation and those who agree to more treatment hours, are more likely to have higher levels of motivation, time and resources to attend assessment and treatment sessions to advocate for their child when services are not being delivered appropriately. Consistent with this theory, Carr et al. (2016) found that families with higher socioeconomic status (a composite variable looking at caregiver occupation and education) had higher levels of treatment attendance.

Among older children (≥5 years of age) who initiated BHT, those with psychiatric and genetic comorbidities, sleep disorders, and those who used antidepressant/antianxiety medications were significantly less likely to continue treatment. It is possible that these co-occurring conditions contribute to greater stress for parents and families, making it harder for families to access care. It has been suggested that higher parent stress contributes to poorer outcomes in children treated with intensive behavioral intervention (Shine and Perry 2010). However, how parental stress contributes to continuation of treatment over time is not known. One study regarding engagement in psychotherapy for children (without ASD) who had behavioral disorders showed that maternal distress was positively associated with dropping out of therapy (Fernandez and Eyberg 2009). Another study looking at parent stress indicated that parents with higher levels of stress actually had improved treatment attendance (Carr et al. 2016), suggesting that other factors in addition to parent stress may play a role in treatment continuation. Regarding co-occurring mental health conditions specifically, there is limited information concerning their impact on engagement in BHT for children with ASD. However, a review of engagement in mental health services for children in general indicates that children with mental health conditions associated with stress or crisis may be more likely to initially engage in psychotherapy but are also more likely to discontinue services prematurely, perhaps due to family psychosocial difficulties/difficulties with family dynamics (Gopalan et al. 2010). For those being treated with medications for psychiatric and medical conditions, parents may be hoping the medication will address their child’s needs and have less motivation to continue BHT. Family members may also have their own psychiatric and medical co-occurring conditions (Karst and Van Hecke 2012) that make pursuing and attending treatment more challenging. Sleep problems have been cited as reasons for families missing therapy sessions (Carr and Lord 2016). It is possible that families of children with genetic syndromes may see the condition as more immutable and therefore may be less likely to continue with intensive, expensive services.

To our knowledge, this study is the first to investigate child and family demographic and clinical characteristics associated with BHT initiation and continuation among a population of children with ASD recently referred for BHT. Our study population was drawn from a large and diverse insured target population, all of whom theoretically had equal access to BHT. Information on outcomes (completion of BHT assessment and treatment delivered) was derived from insurance claims data and therefore was not prone to respondent bias or misclassification. Similarly, all information on demographic and clinical characteristics was retrieved from medical records and not subject to recall bias.

In spite of these strengths, a number of limitations should be noted. The study population was comprised solely of insured patients receiving care in one large health system in northern California, and therefore may not be representative of the treatment experiences of patients covered by alternate insurance providers, or the experiences of the uninsured, and findings may not be generalizable beyond children living in northern California. All information on ASD services was derived from KPNC databases which only record services that are covered and paid for by KPNC. Therefore, there may be missing information about BHT services provided prior to KPNC enrollment, use of speech/language and occupational therapies, and medications not covered by KPNC. Data were incomplete or not available for a number of potentially important predictors of treatment initiation and continuation, such as parental age, education, income, and family composition. Coordinating and managing ABA treatment services can be demanding on the household and can impinge on family resources and quality of life (Karst and Van Hecke 2012). Factors related to time and family resources may better predict whether families have the bandwidth needed to successfully initiate and manage behavioral therapy treatment services.

## Conclusions

In this community based sample of children referred for BHT, only a little more than half remained engaged in BHT 12 months after referral. Factors that contributed to initiation, continuation, and adherence to BHT were younger age at referral, private insurance, more rapid completion of assessments and receiving more than 10 h/week of BHT. In addition, co-occurring psychiatric and medical conditions were related to discontinuation among the older children. Further research into factors that help or hinder families in initiating and continuing BHT is warranted. Additional factors to consider include parent understanding of autism and the reasons their child is being referred for BHT, parent ethnocultural beliefs and attitudes about autism and BHT treatments, parent stress and health status, and parent resources (e.g., financial, time, transportation).
